# Voluntary physical activity counteracts Chronic Heart Failure progression affecting both cardiac function and skeletal muscle in the transgenic Tgαq*44 mouse model

**DOI:** 10.14814/phy2.14161

**Published:** 2019-07-02

**Authors:** Eleonora Bardi, Joanna Majerczak, Jerzy A. Zoladz, Urszula Tyrankiewicz, Tomasz Skorka, Stefan Chlopicki, Magdalena Jablonska, Anna Bar, Krzysztof Jasinski, Alessia Buso, Desy Salvadego, Zenon Nieckarz, Bruno Grassi, Roberto Bottinelli, Maria Antonietta Pellegrino

**Affiliations:** ^1^ Department of Molecular Medicine University of Pavia Pavia Italy; ^2^ Department of Muscle Physiology, Faculty of Rehabilitation University School of Physical Education Krakow Poland; ^3^ Department of Neurobiology Poznan University of Physical Education Poznan Poland; ^4^ Jagiellonian Centre for Experimental Therapeutics (JCET) Jagiellonian University Medical College Krakow Poland; ^5^ Department of Magnetic Resonance Imaging Institute of Nuclear Physics Polish Academy of Sciences Krakow Poland; ^6^ Chair of Pharmacology Jagiellonian University Medical College Krakow Poland; ^7^ Department of Medicine University of Udine Udine Italy; ^8^ Institute of Bioimaging and Molecular Physiology National Research Council Milano Italy; ^9^ Fondazione Salvatore Maugeri (IRCCS) Scientific Institute of Pavia Pavia Italy; ^10^ Interdipartimental Centre for Biology and Sport Medicine University of Pavia Pavia Italy; ^11^ Interuniversity Institute of Myology University of Pavia Pavia Italy

**Keywords:** Chronic heart failure, oxidative stress, voluntary physical activity

## Abstract

Physical activity is emerging as an alternative nonpharmaceutical strategy to prevent and treat a variety of cardiovascular diseases due to its cardiac and skeletal muscle beneficial effects. Oxidative stress occurs in skeletal muscle of chronic heart failure (CHF) patients with possible impact on muscle function decline. We determined the effect of voluntary‐free wheel running (VFWR) in preventing protein damage in Tgαq*44 transgenic mice (Tg) characterized by a delayed CHF progression. In the early (6 months) and transition (12 months) phase of CHF, VFWR increased the daily mean distance covered by Tg mice eliminating the difference between Tg and WT present before exercise at 12 months of age (WT Pre‐EX 3.62 ± 1.66 vs. Tg Pre‐EX 1.51 ± 1.09 km, *P* < 0.005; WT Post‐EX 5.72 ± 3.42 vs. Tg Post‐EX 4.17 ± 1.8 km, *P* > 0.005). This effect was concomitant with an improvement of in vivo cardiac performance [(Cardiac Index (mL/min/cm^2^): 6 months, untrained‐Tg 0.167 ± 0.005 vs. trained‐Tg 0.21 ± 0.003, *P* < 0.005; 12 months, untrained‐Tg 0.1 ± 0.009 vs. trained‐Tg 0.133 ± 0.005, *P* < 0.005]. Such effects were associated with a skeletal muscle antioxidant response effective in preventing oxidative damage induced by CHF at the transition phase (untrained‐Tg 0.438 ± 0.25 vs. trained‐Tg 0.114 ± 0.010, *P* < 0.05) and with an increased expression of protein control markers (MuRF‐1, untrained‐Tg 1.12 ± 0.29 vs. trained‐Tg 14.14 ± 3.04, *P* < 0.0001; Atrogin‐1, untrained‐Tg 0.9 ± 0.38 vs. trained‐Tg 7.79 ± 2.03, *P* < 0.01; Cathepsin L, untrained‐Tg 0.91 ± 0.27 vs. trained‐Tg 2.14 ± 0.55, *P* < 0.01). At the end‐stage of CHF (14 months), trained‐Tg mice showed a worsening of physical performance (decrease in daily activity and weekly distance and time of activity) compared to trained age‐matched WT in association with oxidative protein damage of a similar level to that of untrained‐Tg mice (untrained‐Tg 0.62 ± 0.24 vs. trained‐Tg 0.64 ± 0.13, *P* > 0.05). Prolonged voluntary physical activity performed before the onset of CHF end‐stage, appears to be a useful tool to increase cardiac function and to reduce skeletal muscle oxidative damage counteracting physical activity decline.

## Introduction

It is now widely accepted that chronic physical activity is beneficial for cardiac health emerging as an alternative nonpharmaceutical strategy to prevent and treat a variety of cardiovascular diseases, since it promotes a broad range of cardiac and skeletal muscle beneficial effects (Ventura‐Clapier et al., 2007; Hirai et al., 2015).

Beyond overt cardiovascular alterations, patients with chronic heart failure (CHF) also show exercise intolerance (Poole et al., 2012; Hirai et al., 2015), that affects the patients’ clinical picture, quality of life, and prognosis. Several evidences indicate that changes in cardiac function cannot fully explain the origin of exercise intolerance (Piepoli et al., 2010) and a key role of the periphery has emerged, indicating that changes in skeletal muscle can significantly limit exercise capacity in these patients. The impairment also precludes these patients from obtaining the beneficial effects associated with exercise training (Hirai et al., 2015). Therefore, to improve the quality of life of these patients the development of countermeasures that positively affect both cardiac and skeletal muscle is essential.

Different training modalities are available to target the problems with which chronic heart failure patients are faced and low stress exercise is now recommended for these patients (Aronow, 2003; Piña et al., 2003; Smart and Marwick, 2004).

Voluntary wheel running in rodents is used as a paradigm to assess physical performance and endurance and also to simulate endurance training in humans. The nonforced voluntary wheel running represents a less stressful and more respectful of circadian rhythm intervention than forced running, (Sexton, 1995) able to provide sufficient stimuli for cardiac adaptation (Allen et al., 2001; Natali et al., 2001). Recently, several evidences indicate that voluntary physical activity (free wheel running) has a positive impact on cardiac function in animal models of CHF (Deloux et al., 2017; Grassi et al., 2017; Tyrankiewicz et al., 2017) but it fails to normalize the derangement of skeletal muscle oxidative metabolism associated with the disease (Schultz et al., 2013; Grassi et al., 2017) leaving doubts about the efficacy of free wheel running on skeletal muscle of cardiopathic subjects.

Considering that in healthy conditions, long‐term voluntary wheel running increases oxidative enzymes levels and PGC‐1α specifically in fast skeletal muscles (Ikeda et al., 2006), this could be the reason for its ineffectiveness in affecting skeletal muscle oxidative metabolism in soleus muscle of mice with CHF (Grassi et al., 2017).

Moreover, in addition to metabolic alterations, CHF induces several other alterations in skeletal muscle that include change in content and activity of key radical scavenger enzymes, leading to an increased oxidative stress (Linke et al., 2005; Coirault et al., 2007; Bowen et al., 2015). It is well‐known that under oxidative stress conditions, as in muscles with CHF, the excessive production of reactive oxygen species (ROS) promotes oxidative damage at the proteins level, that is, protein carbonylation, altering tissue homeostasis. In this context, a deleterious effect on in vitro mechanical function due to the myosin carbonylation in skeletal muscle of rats with CHF has been reported (Coirault et al., 2007). Within cells, proteins are under constant surveillance by the proteolytic systems that promote the degradation of proteins damaged by highly reactive molecules, thus operating a protein quality control and preserving muscle health (Kaushik and Cuervo, 2015). Interestingly, reduced oxidative stress (Škop et al., 2015) and increase in proteasome‐mediated proteolysis has been found following both acute (Pasiakos et al., 2010) and chronic endurance exercise (Cunha et al., 2012b) in healthy conditions.

Whether voluntary wheel running is effective in preventing or normalizing oxidative stress and at what stage of the CHF it shows its effectiveness is still unknown. In this study we aimed to determine the impact of voluntary exercise (1) on molecular parameters involved in oxidative metabolism and (2) on normalization of the redox status in the fast gastrocnemius muscle, at different times during the progression of the disease. We hypothesize that voluntary exercise, thanks to its ability to induce catabolic systems activation, promotes an antioxidant effect on skeletal muscle favoring the elimination of oxidized damaged proteins and thus improving physical activity level of cardiopathic mice.

In order to do so, we utilized the Tgαq*44 mice, a transgenic mouse model characterized by a delayed progression from cardiac hypertrophy to overt CHF similar to that described (although over a substantially longer time period) in human patients (Braunwald, 2013), and voluntary‐free wheel running.

## Materials and Methods

### Ethical approval

All animal experiments were conducted according to the National Institutes of Health Guide for the Care and Use of Laboratory Animals and were approved by the local animal protection committee (Local Bioethics Committee in Kraków, approvals No. 914/2012 and 27/2014). The investigators understand the ethical principles under which this journal operates and confirm that the work complies with the journal animal ethics checklist.

### Study design and animal care

The experiments were carried out at three time points, corresponding to different stages of the developing CHF: *4 months of age*, at which mice exhibited no functional impairments (early phase of CHF), *10 months of age*, that is, in mice with impairments of cardiac contractile and mitochondrial functions (transition phase of CHF) and *12 months of age*, at which mice exhibited signs of end‐stage heart failure (end‐stage of CHF). Adult female FVB wild‐type (WT) and homozygous Tgαq*44 transgenic (Tg) mice from several litters were mixed and divided into three age groups (4 months old; 10 months old; 12 months old at the start of the study) and randomized to either sedentary (WT‐CTRL *n* = 11, Tg‐CTRL *n* = 11) or trained group (WT‐T *n* = 11, Tg‐T *n* = 11) for each age group. The analyses were performed in a blinded manner. The training groups were placed in cages equipped with a running wheel, allowing to record the animals’ voluntary wheel running activity during a period of 8 weeks. The animals utilized in this study were bred at the Institute of Experimental and Clinical Medicine of the Polish Academy of Sciences in Warsaw (Poland). Prior to the experiments the animals were transported to the animal house at the Faculty of Pharmacy, Medical College, Jagiellonian University in Krakow (Poland). Mice were housed one per cage (floor area of 355 × 235 × 190 mm) and maintained at 22–24°C under a 12‐h light cycle with ad libitum access to water and rodent chow. The training groups were placed in cages equipped with a running wheel allowing to perform voluntary running activity (see below). For skeletal muscle analysis, the animals of WT‐CTRL, Tg‐CTRL, and Tg‐T groups were killed at 24 h after the last running activity (T groups) by cervical dislocation after euthanasia with ketamine and xylazine (100 and 10 mg/kg intraperitoneal injection, respectively). Gastrocnemius was dissected, immediately frozen in liquid nitrogen and stored at −80°C. Molecular analysis was performed on 4–5 muscle samples for each group.

### Running wheel activity

After a familiarization period with the wheel, voluntary wheel running activity of each mice was recorded continuously using the Running Wheel System (Columbus Instruments Inc., Ohio). The system was programmed to record all running episodes that lasted more than 10 sec. Moreover, the mice were monitored by a digital camera placed in the animal house, allowing the supervising person to check the mice behavior at a given time without disturbing its normal life. Based on the number of revolutions of the wheel and its radius, the covered distance and the running velocity of the animals were calculated. The data were stored in a computer and were downloaded on a weekly basis. The individual data of covered distance and velocity of running were expressed as a mean ± SD value per 24 h, and were further averaged for the entire period of training (56 days).

### Magnetic resonance in vivo study

In vivo cardiac performance was assessed using a 9.4T MRI system (BioSpec, Bruker BioSpin, Ettlingen, Germany) with protocols as described previously (Wojewoda et al., 2016). Briefly, 2D Intragate™ FLASH sequence was used (TE = 1.49 ms; TR = 4.3 ms; flip angle, 18°; number of repetitions, 250; field of view, 30 × 30 mm^2^; matrix size, 256 × 256; slice thickness, 1.0 mm). Image reconstruction was made using the IntraGate 1.2.b.2 macro (Bruker) into a 60‐frame‐per‐cardiac‐cycle movie (or 30 in case of left atrium analysis). Short‐axis views covering the entire left ventricle (LV) were used for LV assessment. Segment package (Heiberg et al., 2010) (v.1.9 R2626, Medviso AB, Lund, Sweden) was used for the LV delineation. The end‐diastolic/end‐systolic volumes (EDV/ESV), ejection fraction [EF = (EDV – ESV)/EDV], cardiac output (CO = SV × HR, where HR is heart rate) were assessed directly using resulting time‐volume (TVC) curve. Left atrial (LA) function was assessed from three long‐axis views at the end of systole and diastole (Tyrankiewicz et al., 2016). Mice were examined under gas anesthesia: 1.7% of isoflurane (Aerrane, Baxter, Deerfield, IL) with oxygen and air in relation 1:2 and under stabilized temperature, maintained at 37°C. During MRI protocols following parameters were monitored: electrocardiogram (R‐R peak intervals), respiration (frequency), and body temperature (maintained at 37°C by water jacket heating). For physiological parameter the tracking Monitoring and Gating System (Model 1025, SA Inc., Stony Brook, NY) was used. Due to the fact that some animals did not present satisfactory ECG signal, which is needed for proper MRI triggering, the number of animals indicated in Table 1 is lower.

**Table 1:** Cardiac performance in Tgαq*44 mice before and after 2 months of spontaneous activity

	Tg‐6mo		Tg‐12mo		Tg‐14mo	
	Tg‐CTRL (*n* = 7)	Tg‐T (*n* = 11)	Tg‐CTRL (*n* = 7)	Tg‐T (*n* = 10)	Tg‐CTRL (*n* = 7)	Tg‐T (*n* = 13)
Body mass	25.8 ± 0.5	24.9 ± 0.5	29.5 ± 3.0^+^	28.6 ± 1.3	29.2 ± 0.7^+^	28.5 ± 0.4
CI [mL/min/cm^2^]	0.167 ± 0.005	0.21 ± 0.003*	0.101 ± 0.009	0.133 ± 0.005*	0.116 ± 0.005	0.119 ± 0.005
HR [bpm]	491 ± 9	499 ± 3	348 ± 14^+^	361 ± 9	321 ± 7^+^	321 ± 6
ESV [μL]	20.0 ± 0.9	25.0 ± 1.3*	31.9 ± 3.2	29.9 ± 1.3	32.5 ± 3.5^+^	30.1 ± 3.8
EDV [μL]	49.2 ± 1.1	60.3 ± 1.6*	59.3 ± 3.4	63.7 ± 2.0	65.1 ± 4.7^+^	62.6 ± 4.4
SV [μL]	29.2 ± 0.7	35.2 ± 0.7*	27.4 ± 2.4	33.7 ± 1.1*	32.6 ± 2.0	32.5 ± 1.1
EF [%]	59.5 ± 1.4	58.7 ± 1.4	46.5 ± 4.4^+^	53.0 ± 1.1*	51.5 ± 2.9	53.6 ± 3.2
ER [SV/RR]	2.91 ± 0.13	3.15 ± 0.10*	3.65 ± 0.38	4.03 ± 0.24*	4.02 ± 0.18^+^	3.99 ± 0.10
FR [SV/RR]	4.01 ± 0.15	3.75 ± 0.10	5.11 ± 0.42	5.71 ± 0.62	5.61 ± 0.46^+^	5.29 ± 0.32
ET [%RR]	39.5 ± 1.4	32.3 ± 1.0*	30.0 ± 2.2	27.0 ± 1.8*	27.4 ± 1.4^+^	25.9 ± 0.9
IVRT [%RR]	13.0 ± 0.7	14.7 ± 0.8*	9.2 ± 2.3	11.9 ± 0.9	11.2 ± 0.9	10.5 ± 0.6
FT [%]	34.5 ± 1.8	37.7 ± 1.2	28.1 ± 4.0	27.5 ± 1.9	25.2 ± 2.4^+^	25.8 ± 2.9
IVCT [% RR]	12.9 ± 0.6	15.2 ± 0.7	32.7 ± 5.0^+^	33.5 ± 2.4	36.1 ± 2.9^+^	37.6 ± 2.7
LA EF [%]	0.16 ± 0.03	0.23 ± 0.01*	0.10 ± 0.04	0.14 ± 0.02	0.05 ± 0.01^+^	0.06 ± 0.01

CI, Cardiac Index; CHF, chronic heart failure; HR, Heart Rate; ESV, End Systolic Volume; EDV, End Diastolic Volume; SV, Stroke Volume; EF, Ejection Fraction; ER, Ejection Rate; FR, Filling Rate (ER and FR were normalized to individual SV and R‐R values); ET, Ejection Time; IVRT, Isovolumic Relaxation Time; FT, Filling Time; IVCT, Isovolumic Contraction Time; LAEF, Left Atrial Ejection Fraction.

Cardiac parameters of transgenic mice at different stages of CHF;

* Significantly different from untrained Tg mice (Tg‐CTRL) at given age (*P *<* *0.05, *t*‐test);

^+^ Significantly different between transgenic mice (Tg‐CTRL) at a given age as compared to 6 months old Tg‐CTRL mice (*P *<* *0.05; One‐way ANOVA); No differences were detected between Tg‐CTRL at the age of 12 and 14 months of age.

### Analysis of MHC isoform content

The myosin heavy chain (MHC) isoform composition was determined using an electrophoretic approach previously described in detail (Brocca et al., 2010). Frozen muscles were pulverized in a steel mortar with liquid nitrogen to obtain a powder that was immediately re‐suspended in Laemmli solution (62.5 mmol/L Tris‐HCl pH 6.8, 2.3% SDS, 10% glycerol, 5% β‐mercaptoethanol, and 0.01% bromophenol blue). Protein concentration was determined with a protein assay kit (RC DC; Bio‐Rad, Hercules, CA). The lysates were loaded onto 8% SDS‐PAGE polyacrylamide gels and electrophoresis was run for 2 h at 200 V and then for 24 h at 250 V. Following Coomassie staining, four bands corresponding to MHC isoforms were obtained and a densitometric analysis was performed to assess the relative proportion of MHC isoforms in the samples.

### Gene expression analysis

Total RNA was extracted from muscles using an SV Total RNA isolation kit (Promega, Madison, WI). The RNA concentration was measured using a NanoDrop instrument (Thermo Scientific, Waltham, MA) and 300 ng was used to generate cDNA with Super‐Script III reverse transcriptase (Invitrogen, Carlsbad, CA). The cDNA was analyzed by quantitative real‐time PCR (AB 7500) using a SYBR Green PCR kit (Applied Biosystems, Foster City, CA) and the data were normalized to PPIA (peptidylprolyl isomerase A) expression (Cannavino et al., 2015b). The oligonucleotide primers used were as follows: Atrogin‐1 Forward GCAAACACTGCCACATTCTCTC Reverse CTTGAGGGGAAAGTGAGACG; MuRF‐1 Forward ACCTGGTGGAAAACATC Reverse CTTCGTGTTCCTTGCACATC; Cathepsin L Forward GCGCGTGACTGGTTGAG Reverse AAAGGCAGCAAGGATGAGTG.

### Western blot analysis

As previously described (Cannavino et al., 2015a), frozen muscle samples were pulverized in a steel mortar with liquid nitrogen and suspended in a lysis buffer (20 mmol/L Tris‐HCl, 1% Triton X‐100, 10% glycerol, 150 mmol/L NaCl, 5 mmol/L EDTA, 100 mmol/L NaF, and 2 mmol/L NaPPi supplemented with 1 × protease, phosphatase inhibitors [(Sigma‐Aldrich, St Louis, MO) and 1 mmol/L phenylmethane sulphonyl fluoride (PMSF)]. The lysate was left for 20 min in ice then centrifuged at 18,000*g* for 20 min at 4°C. Protein concentration was determined on the supernatant using the RC DCTM protein assay kit (Bio‐Rad). Samples were stored at –80°C until ready to use. 40 µg of proteins was loaded onto 12% gradient precast gels (Any kD Mini‐PROTEAN TGX; Bio‐Rad). Proteins were electrotransferred to polyvinylidene difluoride membranes at 100 V for 2 h at 4°C in a transfer buffer containing 25 mmol/L Tris, glycine 192 mmol/L, and 20% methanol. The membranes were probed with specific primary antibodies and subsequently with HRP‐conjugated secondary antibody. Protein bands were visualized by an enhanced chemiluminescence method using a digital imaging system (ImageQuant LAS 4000; GE Healthcare, Litle Chalfont, UK). Protein bands were normalized on actin stained with Ponceau Red. To allow the comparison of samples from different membranes a calibrator sample was loaded in each membrane. The activity levels of AMPKα (5' adenosine monophosphate‐activated protein kinase) were calculated as the ratio between the content in the phosphorylated (p) and total forms.

The primary antibodies used were as follows: rabbit p‐AMPK (thr 172) (Cell Signaling); rabbit AMPK (Cell Signaling); rabbit PGC‐1α (Abcam); mouse OXPHOS complexes (Abcam); rabbit DRP1 (Cell Signaling); rabbit SOD1 (Abcam); rabbit Catalase (Abcam); rabbit LC3B (Sigma‐Aldrich); and anti‐rabbit IgG (Cell Signaling).

### Oxyblot analysis

Muscle samples previously stored at −80°C were pulverized and homogenized at 4°C in an antioxidant buffer containing protease inhibitors, 25 mm imidazole and 5 mm EDTA, pH 7.2 adjusted with NaOH as previously described in detail (Brocca et al., 2010). The lysate was left for 20 min in ice and the homogenate obtained was centrifuged at 18,000*g* for 20 min at 4°C. Protein concentration was determined on the supernatant using the RC DCTM protein assay kit (BioRad). The supernatant was stored at −80°C until ready to use. The protein carbonylation level was detected using the OxyBlot Kit (AbNova, Taipei City, Taiwan), which provides reagents for sensitive immunodetection of these carbonyl groups. The carbonyl groups in the protein side chains are derivatized to 2,4‐dinitrophenylhydrazone (DNP hydrazone) by reaction with 2,4‐dinitrophenylhydrazine (DNPH); 12 μg of the DNP‐derivatized protein samples were separated by PAGE (12% SDS‐polyacrylamide gels) and then blotted for 2 h at 100 V to a nitrocellulose membrane. Membranes obtained were stained with Ponceau Red and then scanned. Membranes were incubated with primary antibody, specific to the DNP moiety of the proteins and subsequently with an HRP–antibody conjugate directed against the primary antibody (secondary antibody: goat anti‐rabbit IgG). Blots were developed using an enhanced chemiluminescence method. Positive bands emitting light were detected by short exposure to photographic films. Protein oxidation was quantified by defining the oxidative index (OI), that is, the ratio between densitometric values of the oxyblot bands and those stained with Ponceau Red.

### Statistical analysis

Data were tested for normal distribution by the Kolmogorov–Smirnov test. Data are represented as mean values ± SD. Statistical analysis was evaluated using the GraphPad Prism software (GraphPad Software) and assessed by a Student’s *t*‐test between Trained and Control mice (*P* < 0.05) at given age. Difference between transgenic mice (Tg‐CTRL) at a given age were assessed by One‐way ANOVA followed by Tuckey’s multicomparisons test or Kruskal–Wallis nonparametric test (*P* < 0.05). Two‐way ANOVA followed by Bonferroni post hoc test was used for comparisons of molecular data between groups at different stages of CHF (*P* ˂ 0.05).

## Results

### In vivo functional performance before and after 2 months of voluntary exercise

Functional performance was evaluated by measuring the total covered distance (km) and the total time (h) spent running during 2 months of voluntary‐free wheel running (VFWR).

**Figure 1:**
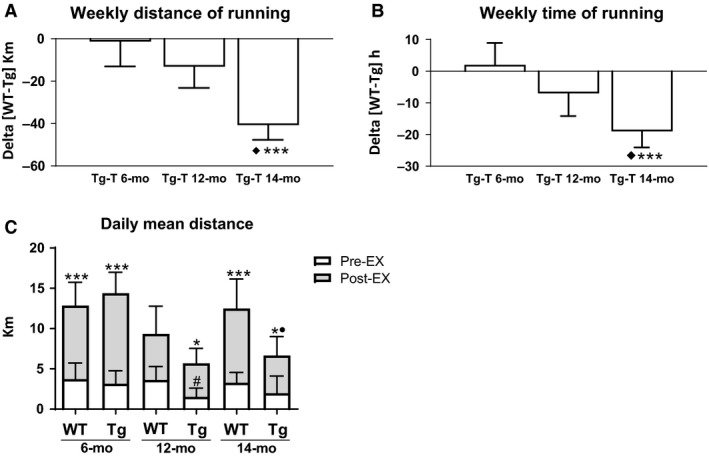
In vivo functional performance. Delta of variation of (A) weekly distance of running (km) and (B) weekly time of running (h) between wild‐type (WT) and transgenic (Tg) trained mice. Star marks show significant difference between Tg‐T and age‐matched WT‐T ***P < 0.001. ♦significant different from Tg‐T 6‐mo and 12‐mo *P* < 0.05; (C) daily mean distance (km) covered by WT and Tg mice before (Pre‐EX) and after (Post‐EX) 2 months of voluntary wheel running at different stages of heart failure. Data are shown as mean ± SD. Star marks show significant difference between Pre‐EX and Post‐EX *P < 0.05 ***P < 0.001. ●significant different from WT Post‐EX 14 months *P* < 0.05. # significant different from WT Pre‐EX 12 months *P* < 0.05.

Tg‐T mice showed a decline in physical activity only in the late phase of the disease. In fact, at 14 months of age both weekly distance [delta (WT‐T‐Tg‐T) − 40.72 km] and time [delta (WT‐T‐Tg‐T) − 19 h] spent on the wheel (Fig. 1A and B) were significantly lower in Tg‐T with respect to the age‐matched WT‐T. Both weekly distance and time of running of Tg‐T at 6 and at 12 months of age were not significantly different from their age‐matched WT‐T. The comparison of daily covered distance before (Pre‐EX) and after (Post‐EX) 2 months of VFWR, showed a significant improvement of physical activity level in Tg mice (Fig. 1C) at all ages. VFWR eliminated the difference in daily performance between Tg and WT present before exercise at 12 months of age (WT Pre‐EX 3.62 ± 1.66 km vs. Tg Pre‐EX 1.51 ± 1.09 km, *P* < 0.005; WT Post‐EX 5.72 ± 3.42 km vs. Tg Post‐EX 4.17 ± 1.8 km, *P* > 0.005).

Although the positive effect of exercise at all stage of the disease, at 14 months of age a significant functional gap between WT and Tg mice was observed, as indicated by the significant decrease in weekly distance (Fig. 1A) and time (Fig. 1B) and daily distance (Fig. 1C) of running.

### Cardiac performance before and after 2 months of voluntary exercise

Results related to cardiac performance in Tg mice are presented in Table 1. Parameters related to cardiac function were found altered in 12 and 14 months Tg‐CTRL old mice compared to those of Tg‐CTRL at 6 months of age. More precisely, a significant decreased of ejection fraction (EF), heart rate (HR), and prolonged sovolumic contraction time (IVCT) was found at the age of 12 months. Further deterioration was observed in 14 months‐old mice as decreases in left atrial ejection fraction (LA EF), increases in end systolic volume (ESV), end diastolic volume (EDV), ejection rate (ER) and filling rate (FR), reduction of ejection time (ET), filling time (FT), and heart rate (HR) and further prolongation of IVCT. No differences were detected between Tg‐CTRL at the age of 12 and 14 months of age.

Spontaneous activity improved cardiac performance in mice at the early and at the transition phase of CHF by increasing Cardiac Index (CI) (*at 6 months*, Tg‐CTRL 0.167 ± 0.005 vs. Tg‐T 0.21 ± 0.003 *P* < 0.05; *at 12 months*, Tg‐CTRL 0.101 ± 0.009 vs. Tg‐T 0.133 ± 0.005 *P* < 0.05), Stroke Volume (SV) (*at 6 months*, Tg‐CTRL 29.2 ± 0.7 vs. Tg‐T 35.2 ± 0.7 *P* < 0.05; *at 12 months*, Tg‐CTRL 27.4 ± 2.4 vs. Tg‐T 33.7 ± 1.1 *P* < 0.05), ER (*at 6 months*, Tg‐CTRL 2.91 ± 0.13 vs. Tg‐T 3.15 ± 0.1 *P* < 0.05; *at 12 months*, Tg‐CTRL 3.65 ± 0.38 vs. Tg‐T 4.03 ± 0.24 *P* < 0.05), and by shortening ET (*at 6 months*, Tg‐CTRL 39.5 ± 1.4 vs. Tg‐T 32.3 ± 1 *P* < 0.05; *at 12 months*, Tg‐CTRL 30 ± 2.2 vs. Tg‐T 27 ± 1.8 *P* < 0.05). At the early phase of CHF spontaneous activity additionally significantly increased ESV (Tg‐CTRL 20 ± 0.9 vs. Tg‐T 25 ± 1.3 *P* < 0.05), EDV (Tg‐CTRL 49.2 ± 1.1 vs. Tg‐T 60.3 ± 1.6 *P* < 0.05) and LA EF (Tg‐CTRL 0.16 ± 0.03 vs. Tg‐T 0.23 ± 0.01 *P* < 0.05), and prolonged Isovolumic Relaxation Time (IVRT) (Tg‐CTRL 13 ± 0.7 vs. Tg‐T 14.7 ± 0.8 *P* < 0.05).

### In vitro skeletal muscle determinations before and after 2 months of voluntary exercise

In order to understand the efficacy of voluntary exercise in normalizing molecular parameters associated with CHF, the WT‐CTRL, Tg‐CTRL, and Tg‐T groups were analyzed as follows:

### MHC isoforms composition before and after 2 months of voluntary exercise

**Figure 2:**
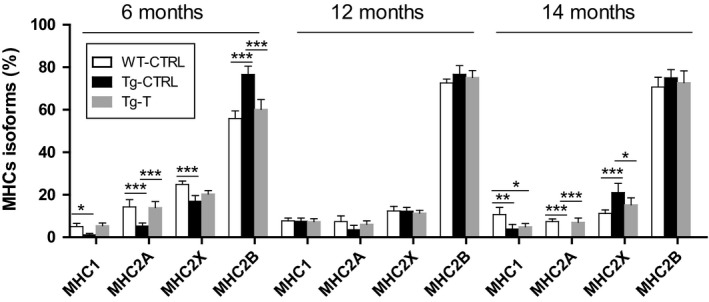
MHC isoforms composition. Myosin heavy chain (MHC) isoforms composition in WT‐CTRL, Tg‐CTRL, and Tg‐T groups, at 6, 12, and 14 months of age that correspond to different stages of heart failure. Data are shown as mean ± SD. *P < 0.05, **P < 0.01, ***P < 0.001.

MHC isoform composition was determined as an index of fiber‐type composition. At 6 months of age CHF induced a significant slow‐to‐fast shift in MHC composition, as indicated by the significantly higher fast MHC‐2B content, with concomitant significantly lower contents of MHC‐1 and MHC‐2A, observed in Tg‐CTRL in comparison with WT‐CTRL (Fig. 2). Exercise training induced a significant reduction in MHC‐2B and a concomitant increase in MHC‐1 and MHC‐2A, in comparison with Tg‐CTRL. No significant effects of CHF and exercise training on MHC isoform content were found at 12 months of age. At 14 months of age Tg‐CTRL mice showed a substantial reduction in MHC‐2A and MHC‐1, with a concomitant significant increase in MHC‐2X with respect to WT‐CTRL. Exercise training restored the control phenotype at 6 months of age and partially at 14 months.

### Antioxidant enzymes and protein carbonylation before and after 2 months of voluntary exercise

**Figure 3:**
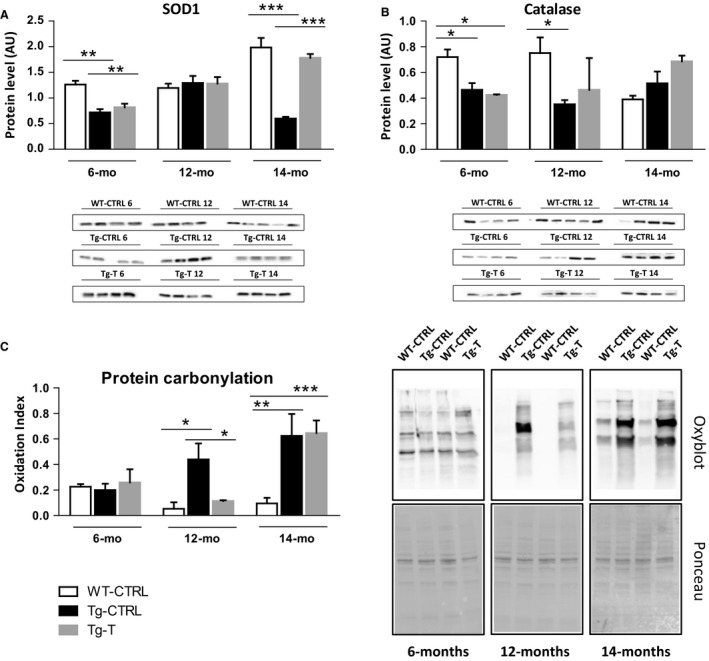
Antioxidant defense system. Quantification of (A) SOD1(superoxide dismutase 1) protein level and (B) catalase protein level by western blot analysis (C) proteins carbonylation by Oxyblot analysis in WT‐CTRL, Tg‐CTRL, and Tg‐T mice, at 6, 12, and 14 months of age that correspond to different stages of heart failure. Data are shown as mean ± SD *P < 0.05 **P < 0.01 ***P < 0.001.

Since an increase in ROS production has been demonstrated in muscles of CHF patients, we studied the development of redox imbalance during the progression of the disease by evaluating the protein level of two antioxidant enzymes, superoxide dismutase 1 (SOD1) and catalase, and the level of protein carbonylation. In Tg‐CTRL mice a significantly lower SOD1 content was found at 6 and 14 months of age, in comparison to the WT‐CTRL group. Exercise training significantly counteracted SOD1 reduction only in the late stage of the disease (14 months of age) (Fig. 3A). Catalase protein level was found significantly reduced at 6 and 12 months of age in Tg‐CTRL mice versus WT‐CTRL, and its expression was not significantly modified by exercise training at any age (Fig. 3B). Protein carbonylation was significantly higher in Tg‐CTRL versus WT‐CTRL at 12 (0.438 ± 0.25 vs. 0.053 ± 0.1 *P* < 0.05) and 14 (0.623 ± 0.24 vs. 0.095 ± 0.087 *P* < 0.01) months of age. Exercise prevented oxidative protein damage only at 12 months of age (Tg‐CTRL 0.438 ± 0.25 vs. Tg‐T 0.114 ± 0.01 *P* < 0.05) (Fig. 3C).

### Protein quality control system before and after 2 months of voluntary exercise

**Figure 4:**
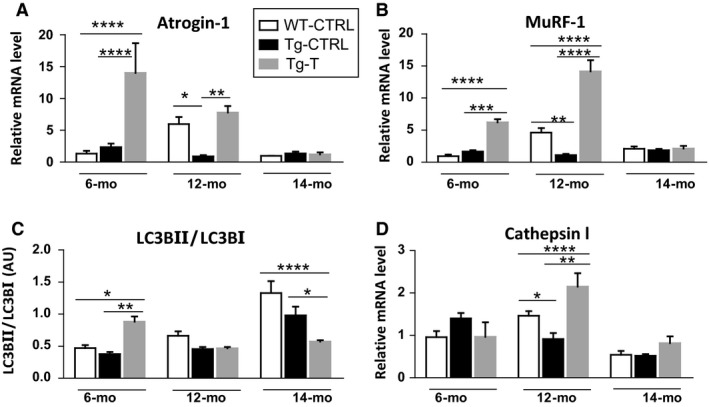
Protein quality control systems. Quantification of mRNA levels of (A) Atrogin‐1, (B) MuRF‐1 (muscle‐specific ring finger protein‐1) and (D) cathepsin L by RT‐PCR (C) protein level quantification of LC3 (microtubule‐associated protein light chain 3) based on the ratio between the content in forms II and I of LC3 by western blotting in WT‐CTRL, Tg‐CTRL, and Tg‐T mice, at 6, 12, and 14 months of age that correspond to different stages of heart failure. Data are shown as mean ± SD. *P < 0.05, **P < 0.01, ***P < 0.001, ****P < 0.0001.

As the ubiquitine–proteasome system and autophagy are the major systems involved in protein quality control, we measured the mRNA levels of the muscle‐specific ubiquitin E3‐ligases, Atrogin‐1, and MuRF‐1, which belong to ubiquitine–proteasome system, and LC3 protein level and Cathepsin‐L mRNA levels as markers of the autophagy machinery. In the intermediate phase of CHF (12 months of age) Tg‐CTRL showed significantly lower mRNA levels of Atrogin‐1 and MuRF‐1, with respect to WT‐CTRL (Fig. 4A and B). In Tg‐T, Atrogin‐1 and MuRF‐1 levels increased with training with respect to Tg‐CTRL (*at 6 months*, MuRF‐1, Tg‐CTRL 1.65 ± 0.47 vs. Tg‐T 6.2 ± 0.99 *P* < 0.001; Atrogin‐1, Tg‐CTRL 2.34 ± 1.11 vs. Tg‐T 14.02 ± 9.3 *P* < 0.0001; *at 12 months*, MuRF‐1, Tg‐CTRL 1.12 ± 0.29 vs. Tg‐T 14.14 ± 3.04 *P* < 0.0001; Atrogin‐1, Tg‐CTRL 0.9 ± 0.38 vs. Tg‐T 7.79 ± 2.03 *P* < 0.01), with the exception of the late stage of the disease (14 months) (Fig. 4A and B).

As regards markers of autophagy, LC3II/LC3I was not affected by CHF at all stages of the disease (Fig. 4C). Cathepsin‐L was found significantly increased in Tg‐CTRL mice at 6 months, and decreased at 12 months (Fig. 4D). Exercise‐induced autophagy through the increase in LC3II/LC3I at 6 months (Tg‐CTRL 0.377 ± 0.069 vs. Tg‐T 0.883 ± 0.15 *P* < 0.01) and Cathepsin‐L at 12 months (Tg‐CTRL 0.915 ± 0.27 vs. Tg‐T 2.14 ± 0.55 *P* < 0.01). In the late stage of the disease (14 months) exercise training failed to induce autophagy.

### AMPK and its targets before and after 2 months of voluntary exercise

**Figure 5:**
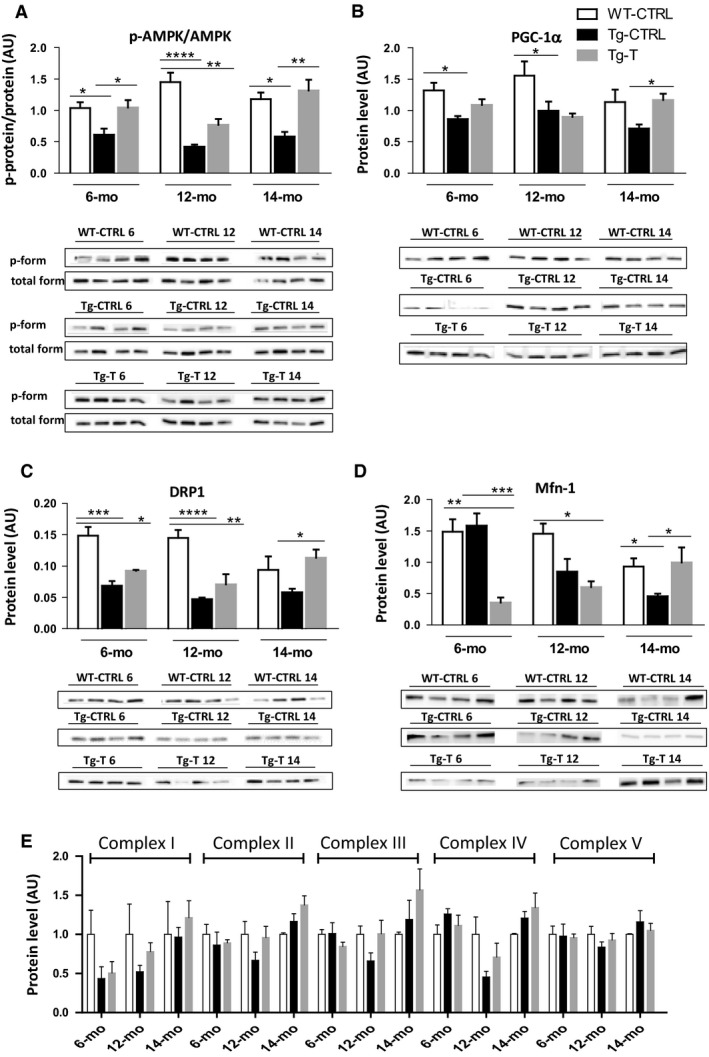
Energetic and oxidative parameters. Quantification of (A) AMPK (AMP‐activated protein kinase) phosphorylation level (B) PGC‐1α (peroxisome proliferative activated receptor‐γ coactivator 1α) protein level (C) DRP1 (dynamin‐related protein 1) protein level (D) Mfn‐1 (mitofusin1) protein level (E) mitochondrial complexes protein levels (I, II, III, IV, V) by western blot analysis in WT‐CTRL, Tg‐CTRL and Tg‐T mice, at 6, 12 and 14 months of age that correspond to different stages of heart failure. The activity level of AMPK was calculated as the ratio between the content in the phosphorylated (p‐form) and total form. Data are shown as mean ± SD. *P < 0.05 **P < 0.01 ***P < 0.001 ****P < 0.0001.

AMPK functions as a central mediator of the cellular response to energetic stress controlling mitochondrial homeostasis (Herzig and Shaw, 2018). Figure 5A shows changes in AMPK activation, evaluated by the ratio between the phosphorylated and total AMPK (p‐AMPK/AMPK). In Tg‐CTRL, at all stages of disease, p‐AMPK/AMPK was significantly lower with respect to WT‐CTRL. In Tg‐T exercise induced a significant p‐AMPK/AMPK increase; at 12 months of age the increase was not statistically significant. As shown in panel B, a decrease in PGC‐1α protein content was found in Tg‐CTRL mice with respect to the age‐matched WT‐CTRL mice, in all phases of the disease (although at 14 months of age the difference did not reach statistical significance), suggesting that the basal level of mitochondrial biogenesis was impaired by CHF. Exercise training induced a significant PGC‐1α increase in Tg‐T mice only at 14 months of age.

Since the key to maintaining mitochondrial health is mitochondrial dynamics and AMPK is a crucial regulator of this process, we analyzed the expression of the profission dynamin fission‐related protein 1 (DRP1) and the profusion mitofusin1 (Mfn1). Tg‐CTRL mice showed a significantly lower DRP1 content, with respect to the age‐matched WT‐CTRL mice, in all phases of the disease (panel C). Panel D shows Mfn‐1 protein levels, Tg‐CTRL mice showed lower Mfn‐1 level at 12 and 14 months of age with respect to the age‐matched WT‐CTRL mice. As for PGC‐1α, exercise training significantly counteracted DRP1 and Mfn‐1 reduction only in the late stage of the disease (14 months of age). The protein level of mitochondrial complexes was also evaluated and no significant changes were found among the different experimental groups (panel E).

## Discussion

Tacking advantage of Tgαq*44 mouse model with protracted development of chronic heart failure (CHF), we investigated the effect of voluntary exercise in normalizing skeletal muscle oxidative stress during the progression of CHF.

Voluntary exercise, despite not affecting key molecular parameters related to skeletal muscle oxidative metabolism, turned out an effective stimulus to counteract oxidative stress in skeletal muscle of cardiopathic mice. This positive effect was observed before the onset of the end‐stage of CHF and appears to be mediated by the induction of pathways involved in the protein quality control and associated with improvement of physical activity level and cardiac function.

### Voluntary exercise positively affects physical activity level and cardiac function

The in vivo muscle performance was analyzed by measuring the covered distance and time of activity after 2 months of voluntary wheel running. Availability of the running wheel in the cage determined a significantly increased level of spontaneous activity, both in wild‐type and transgenic mice as indicated by the significant increase in daily distance covered after exercise. This is in agreement with the improved running capacity that has been observed with voluntary wheel running exercise in rats with CHF (Schultz et al., 2013). Voluntary exercise was also able to eliminate the difference in daily performance between wild‐type and cardiopathic mice present before exercise at 12 months of age. Despite the beneficial effect of exercise, transgenic mice showed a functional impairment in the late stage of disease (14 months), as shown by the significant reduction in daily activity and weekly distance and time of running.

In cardiopathic mice at the early stage of the disease the cardiac performance is still preserved (Tyrankiewicz et al., 2017). During the progression of CHF a progressive impairment of ventricular and atrial functions occurred as indicated by the increased number of cardiac parameters compromised in the intermediate and final phases of the disease compared to the early phase. Furthermore, the peripheral consequences of progressing CHF may be observed as more profound impairment in running activity at the finale stage of the disease, in agreement with previous observations showing an evident cardiac decompensation at ~14 months of age, leading to the phenotype of end‐stage heart failure (Elas et al., 2008; Mackiewicz et al., 2012; Czarnowska et al., 2016; Tyrankiewicz et al., 2017).

Exercise training improved ventricular and atrial functions (cardiac index, end systolic volume, end diastolic volume, stroke volume ejection fraction, left atrial ejection fraction) both at the early and transition phase of CHF (6 and 12 months), but not in the advanced state of the disease (14 months of age), indicating that the improved cardiac function, at least in part, contributes to the improved physical activity level found at the early and transition phase of CHF.

### Voluntary exercise protected muscle from CHF‐induced oxidative stress in the transition phase of the disease

Considering the oxidative stress can contribute to skeletal muscle functional impairment (Kuwahara et al., 2010), we investigated these aspects in our animal model during the progression of the disease. In a normal and healthy situation, a basal level of antioxidant enzymes is maintained to protect against deleterious effects of free radicals. The decreased SOD1 and catalase levels found early and at the advanced stages of the disease, provide the basis for ROS accumulation (Brocca et al., 2010). Consistently, a significant increase in protein carbonylation was found both at 12 and 14 months. These results confirm the accepted view that redox imbalance and oxidative stress occur in skeletal muscle of CHF patients/animals (Linke et al., 2005; Coirault et al., 2007; Bowen et al., 2015). Considering that a deleterious effect on in vitro myosin mechanical function due to myosin carbonylation has been reported in skeletal muscle of rats with CHF (Coirault et al., 2007), and that in vivo performance is negatively affected by excessive ROS production (Kuwahara et al., 2010), it is reasonable to hypothesize that in mice with CHF protein carbonylation could be responsible, at least in part, for the development of skeletal muscle dysfunction and physical activity decline.

Regular physical activity can prevent oxidative stress by either increasing the antioxidant defenses of skeletal muscle (Brooks et al., 2008) or normalizing the oxidized proteins level, through induction of systems responsible for their elimination (Pasiakos et al., 2010). In Tgαq*44 mice, exercise was able to ensure normal levels of protein oxidation at 12 but not at 14 months of age, when the damage was presumably too extensive to be overcome by exercise. Carbonylation is an irreversible oxidative process and carbonylated proteins are marked for proteolysis by the proteasome that acts as reactive carbonyl scavenger (see discussion below). Considering that at the beginning of exercise 12‐month‐old transgenic mice are characterized by high level of carbonylated proteins, it is reasonable to think that exercise improves systems responsible for their elimination. Moreover, the absence of carbonylation obtained with exercise in cardiopathic mice in the intermediate phase of the disease (12 months) also suggests that endogenous defense systems, acting as ROS scavenger, are efficient at keeping disease‐induced ROS at the control level. In this context, SOD and catalase do not appear to be responsible for this effect. To effectively neutralize ROS, an increase in expression of both SOD1 and catalase is required. Whereas SOD1 levels were increased by training at 14 months, no effects of training on catalase levels were observed in this study at any time‐point. This suggests that other antioxidant proteins take part in this process. In fact, it is known that free radicals are neutralized by an elaborate antioxidant defense system, including glutathione and numerous nonenzymatic antioxidants (Valko et al., 2007).

Interestingly, the antioxidant effect of exercise found in cardiopathic mice at 12 months was associated with a significant improvement of physical activity that was no different from that of trained wild type mice at the same age. Instead, at the end stage of CHF (14 months), the physical performance was significantly compromised and associated with the accumulation of carbonylated proteins. Thus, voluntary physical activity, has a beneficial effect in countering skeletal muscle oxidative stress in Tgαq*44 mice, but only before the attainment of the end‐stage of CHF.

### The improved physical activity level induced by training was associated with induction of protein quality control systems

Maintenance of muscle performance relies on a baseline turnover of mechanically unfolded and damaged proteins, preventing cytotoxic accumulation of aggregates. Exercise training‐induced skeletal muscle adaptation likely requires both addition and clearance of cellular components. Therefore, it is believed that proteolysis processes play a role in healthy skeletal muscle adaptation and post‐exercise remodeling (Bell et al., 2016). In this study, the ubiquitin–proteasome markers were unaffected by CHF at the early stage of the disease, whereas they decreased in the crucial period of the transition from compensated hypertrophy to decompensated CHF (12 months). A similar trend was observed for autophagic markers. Considering that carbonylated proteins accumulated at 12 and 14 months and that the ubiquitin–proteasome system is involved in their degradation (Davies, 2001), it can be concluded that adaptations of protein quality control systems were not favorable for homeostasis maintenance. Decrease in proteasome activity has been indicated in the diaphragm of rats with CHF (Bowen et al., 2015). Reduction or lack of induction of proteasome and autophagy may both cause and result from increased levels of oxidized proteins. Persistently high ROS levels, in fact, may lower the efficiency of the ubiquitin–proteasome system, as a consequence of its susceptibility to oxidative stress (Bulteau et al., 2001). Therefore, a higher level of oxidative stress, as indicated by the high levels of protein carbonylation, likely impaired protein control systems in Tgαq*44 mice at the age of 12 and 14 months. This conclusion seems to contradict the overactivation of proteasomal activity in presence of protein carbonylation observed in a mouse model of CHF induced by sympathetic hyperactivity (Cunha et al., 2012a; Cunha et al., 2012b). However, it might be hypothesized that between 6 and 12 months of age an overinduction of protein control systems actually occurred in Tgαq*44 mice, preceding the subsequent impairment observed at 12 months.

Several evidences have shown that exercise induces a general upregulation of ubiquitin–proteasome level both in humans (Stefanetti et al., 2015) and mice (Cunha et al., 2012b). Furthermore, autophagy activation in skeletal muscle following a single bout of forced treadmill exercise (Grumati et al., 2011; He et al., 2012), as well as after prolonged voluntary wheel‐running exercise (Lira et al., 2013) has been reported. In agreement with this, in this study exercise training resulted in an upregulation of the ubiquitin proteasome and autophagy markers. Such effect was observed only in the early and transition stages of CHF, in which the in vivo improved functional performance obtained by training was similar to that of the age‐matched WT trained mice and no protein carbonylation occurred.

Collectively, our results indicate that at stages preceding the end‐stage disease, but not at the end‐stage of CHF, exercise training improved the expression of such systems, preventing oxidized proteins accumulation and contributing to maintaining the in vivo performance. These results also confirm that an over‐activation of skeletal muscle proteolytic systems is not restricted to atrophying states and plays a role in maintaining cellular homeostasis.

### Improvement of physical activity level occurred without normalization of molecular factors involved in oxidative metabolism

To verify whether the positive effect of free wheel running on physical activity level was mediated, at least in part, by the correction of muscle metabolic defects associated with CHF, we investigated this aspect during the progression of the disease.

Acting as a major cellular energy sensor, AMPK has a central role in reprogramming cellular metabolism from anabolism to catabolism. This energy switch controls several cellular processes, including mitochondrial homeostasis (Herzig and Shaw, 2018). Tgαq*44 mice showed a basal AMPK signaling suppression, in all phases of the disease. Since PGC‐1α has a prominent role in metabolic adaptations to the energetic status, its activity might be targeted by cellular mechanisms capable to sense perturbations in cellular energy balance. One of these mechanisms is represented by AMPK. Phosphorylation of PGC‐1α by AMPK is, therefore, part of the link between the sensing of the energetic status and the induction of transcriptional programs that control energy expenditure. Decreased PCG‐1α levels have been found in an animal model of heart failure, both in cardiac (Knowlton et al., 2014) and skeletal muscle (Ventura‐Clapier et al., 2004). Accordingly, we found decreased PCG‐1α levels in Tgαq*44 mice starting at the early stage of the disease, also in agreement with the slow to fast MHC isoforms transition. It is, in fact, known that PCG‐1α drives the formation of slow‐twitch muscle fibers (Lin et al., 2002). The data clearly show the AMPK‐ PGC‐1α axis is depressed early and during the progression of CHF in Tgαq*44 mice. It is widely established that AMPK is activated in response to the increased energetic load produced by exercise and AMPK activation in skeletal muscle during exercise improves athletic performance (Jørgensen et al., 2005). Furthermore, it is known a profission responses in mitochondrial dynamics mediated by AMPK (Toyama et al., 2016) and exercise (Trewin et al., 2018) later followed by a profusion adaptive responses. Mitochondrial dynamics is essential for mitochondrial viability and response to changes in cellular bioenergetics status. Change in metabolic demand regulates the rate of fission and fusion causing mitochondria to become either fragmented or hypertubular (Srivastava, 2017). In this study, exercise increased AMPK level which in principle could contribute to the improved physical activity level. However, no increase in several direct or indirect targets of AMPK related to mitochondrial biogenesis and dynamics was observed until the age of 12 months, that is, at times when physical activity level increased, whereas they increased at 14 months, that is, at time when Tg mice develop exercise intolerance. In this context, the restoring of normal muscle phenotype observed in exercised cardiopathic mice did not occur as a function of PGC‐1α content, in agreement with the concept that endurance exercise‐induced fiber‐type transformation in skeletal muscle can occur independently from the function of PGC‐1α (Geng et al., 2010). Accordingly, we can conclude that the keys factors involved in mitochondrial biogenesis and dynamics did not contribute to the improvement of physical activity level induced by voluntary exercise even in fast muscle. It is tempting to speculate that mitochondrial remodeling in skeletal muscles is triggered only when prolonged exercise was not able to improve cardiac function as a last resort to improve physical activity level increasing the cellular oxidation capacity (Herzig and Shaw, 2018).

In summary, the Tgαq*44 mouse model gave us the opportunity to evaluate the effect of prolonged regular exercise on cardiac function, skeletal muscle adaptations and physical activity level during the progression of CHF from the compensated to the uncompensated state of the disease. The training modality used in this study, in which work rate cannot be determined, does not allow us to suggest specific exercise prescriptions to be extrapolated to other animal species or to humans. What we can conclude is that the observed results were obtained by a prolonged voluntary submaximal exercise training intervention that in the early/transition stages of CHF, despite not affecting molecular factors involved in skeletal muscle oxidative metabolism, leads to positive effects not only on cardiac function but also in skeletal muscle reducing oxidative damage and thus representing a useful therapeutic modality to counteract physical activity decline.

## Conflict of Interest

All authors declare no conflicts of interest.
